# Simultaneous Electrochemical Detection of Nitrite and Hydrogen Peroxide Based on 3D Au-rGO/FTO Obtained Through a One-Step Synthesis

**DOI:** 10.3390/s19061304

**Published:** 2019-03-15

**Authors:** Chengcheng Li, Delun Chen, Yuanyuan Wang, Xiaoyong Lai, Juan Peng, Xiaohong Wang, Kexi Zhang, Yang Cao

**Affiliations:** 1 State Key Laboratory of Marine Resource Utilization in South China Sea, College of Materials and Chemical Engineering, Hainan University, Haikou 570228, China; lichengcheng@hainu.edu.cn (C.L.); chendelun2014@163.com (D.C.); wangxiaohong@hainu.edu.cn (X.W.); 2 Laboratory of Tropical Biomedicine and Biotechnology, School of Tropical Medicine and Laboratory Medicine, Hainan Medical University, Haikou 571199, China; vivian1004@126.com; 3 Laboratory Cultivation Base of Natural Gas Conversion, School of Chemistry and Chemical Engineering, Ningxia University, Yinchuan 750021, China; xylai@nxu.edu.cn (X.L.); pengjuan@nxu.edu.cn (J.P.)

**Keywords:** one-step electrodeposition, nitrite sensing, hydrogen peroxide sensing, 3D Au-rGO/FTO, simultaneous detection

## Abstract

In this paper, Au and reduced graphene oxide (rGO) were successively deposited on fluorine-doped SnO_2_ transparent conductive glass (FTO, 1 × 2 cm) via a facile and one-step electrodeposition method to form a clean interface and construct a three-dimensional network structure for the simultaneous detection of nitrite and hydrogen peroxide (H_2_O_2_). For nitrite detection, 3D Au-rGO/FTO displayed a sensitivity of 419 μA mM^−1^ cm^−2^ and a linear range from 0.0299 to 5.74 mM, while for the detection of H_2_O_2_, the sensitivity was 236 μA mM^−1^ cm^−2^ and a range from 0.179 to 10.5 mM. The combined results from scanning electron microscopy (SEM), transmission electron microscopy (TEM), Raman spectroscopy, X-ray diffraction measurements (XRD) and electrochemical tests demonstrated that the properties of 3D Au-rGO/FTO were attributabled to the conductive network consisting of rGO and the good dispersion of Au nanoparticles (AuNPs) which can provide better electrochemical properties than other metal compounds, such as a larger electroactive surface area, more active sites, and a bigger catalytic rate constant.

## 1. Introduction

In recent years, sensors with high selectivity and sensitivity have attracted much attention. Different electrochemical sensors used as vital sensors for the detection of small molecules, have been built via simple methods [1,2]. Actually, the detection of different molecules under the same conditions is a challenge and necessity in practical application as most electrochemical sensors cannot not simultaneously detect multiple targets in the same environment. For example, nitrite and hydrogen peroxide (H_2_O_2_) are of great importance in environmental monitoring, food safety and other fields, but the detection of both species concurrently has attracted little attention due to the degree of difficulty of developing appropriate sensors. To the contrary, the detection of each one of them separately has been widely reported.

According to the literature, Au nanoparticles (AuNPs) or carbon materials have been used to monitor the concentrations of nitrite and H_2_O_2_ separately. For example, the concentration of H_2_O_2_ was detected using electrodes modified with AuNPs developed by Liu et al. [3] and AuNPs/multi-walled carbon nanotubes developed by Feng et al. [4] Meanwhile, AuNPs and AuNPs/multi-walled carbon nanotubes were employed to monitor the concentration of nitrite by Afkhami et al. [5] and Zou et al. [6] respectively. However, the separate detection of nitrite and H_2_O_2_ is not convenient, and the simultaneous detection of nitrite and H_2_O_2_ is expected. An approach for detecting glucose, hydrogen peroxide and nitrite has been developed by Wu et al. [7], indicating that simultaneous detection of nitrite and H_2_O_2_ is feasible with electrodes modified by Au-carbon nanocomposite.

Graphene and AuNPs were discovered to be good alternatives for the sensitive detection of various molecules [8,9,10,11]. Hence, a myriad of methods for Au-GO nanocomposite preparation have been developed. For example, AuNPs/nitrogen-doped graphene quantum dots were prepared by Chen et al. through a hydrothermal method and refluxing process [12]; Au-rGO/ poly(diallyldimethylammonium chloride) (PDDA) nanocomposites were prepared by Wang et al. using PDDA for the reduction and stability in refluxing conditions at 90 °C for 12 h [13]. To sum up, Au-GO nanocomposite with good properties can be synthesized in different ways, but some shortcomings such as complex synthesis steps, long time consumption and the need to utilize surfactant, etc. are found in these methods. Therefore, the development of a simple and rapid method for the synthesis of Au-GO nanocomposites with clean interfaces is essential. Electrochemical deposition is an ideal method. It has been reported in the literature that a series of materials can be synthesized by this method [14,15,16]. In particular, AuNPs/flower-like structure graphene was prepared by Du et al. via a two-step electrodeposition [17].

In the current work, SnO_2_ transparent conductive glass (FTO) surface was coated with 3D Au-rGO by means of a one-step electrodeposition in an electrolyte which contained NaCl, which could help to form a clean interface and construct a three-dimensional network structure. This structure could effectively enhance the transfer of electrons in the reaction process and improve the detection performance. Nitrite and H_2_O_2_ were detected simultaneously by this electrode. In addition, the electroactive surface area, number of active sites, and catalytic rate constant of the electrode were evaluated through electrochemical analysis. A possible mechanism of detection was discussed in this work too.

## 2. Materials and Methods

### 2.1. Chemical Reagents and Equipment

GO was acquired from Nanjing Xianfeng Nano Technology (Nanjing, China), FTO (1 × 2 cm) glass was obtained from Yingkou New Energy Technology (Yingkou, China), disodium hydrogen phosphate (Na_2_HPO_4_) and sodium dihydrogen phosphate (NaH_2_PO_4_) were purchased from Xilong Scientific (Shantou, China), chloroauric acid (HAuCl_4_) was procured from Kema Biochemical (Tianjin, China), glucose, potassium chloride (KCl) and disodium chloride (NaCl) were gained from Guangzhou Chemical Reagent Factory (Guangzhou, China). Ascorbic acid (AA) and uric acid (UA) were obtained from Sinopharm Chemical Reagent (Shanghai, China) and Shanghai McLean Biochemical Technology (Shanghai, China), respectively. All chemicals were of analytical reagent grade and used as received. Phosphate buffer solution (PBS, 0.1 M) with pH 7.5 prepared from NaH_2_PO_4_ and Na_2_HPO_4_ was selected for the electrolyte solution in the present experiments. Double distilled water was used throughout the experimental process.

X-ray diffraction (XRD) analysis was performed using a D8 Tools XRD instrument (Bruker, Karlsruhe, Germany). The Raman spectrum was acquired on an inVia Reflex Laser Raman spectrograph (Renishaw Corporation, Gloucestershire, UK). Field-emission scanning electron microscope (FE-SEM) images were observed using a Hitachi S-4800 instrument (Tokyo, Japan). Transmission electron microscopy (TEM) micrographs were acquired using a JEOL-2100F microscope (Tokyo, Japan). All electrochemical experiments were investigated by a three-electrode system, obtained on an SP-200 Electrochemical Workstation (Bio-Logic Science Instruments, Paris, France), and the modified FTO (3D Au-rGO/FTO, Au/FTO), platinum sheet (2 cm × 2 cm), and saturated Ag/AgCl were used as working, counter, and reference electrodes, respectively.

### 2.2. Preparation of 3D Au–rGO/FTO

3D Au-rGO/FTO was prepared via one-step electrodeposition at room temperature and without stirring. 3D Au-rGO was electrodeposited on the cleaned FTO through cyclic voltammetry (CV) in the potential range from −1.6 V to 0.8 V with 50 cycles. The electrolyte was 5 mM NaCl solution with 50 μg/mL GO and 25 μg/mL HAuCl_4_. For comparison, Au/FTO was prepared under the same condition but the electrolyte without GO.

### 2.3. Electrochemical Activity and the Detection of Nitrite and H_2_O_2_

Firstly, EIS and CVs of Au/FTO and Au-rGO/FTO electrodes were carried out in potassium ferricyanide (K_3_Fe(CN)_6_, 5 mM) solution. The CVs of various concentrations of nitrite and H_2_O_2_ in 0.1 M, pH = 7.5 PBS were implemented using the pre-treated Au–rGO/FTO. Lastly, the Au-rGO/FTO was used to detect the mixture of nitrite and H_2_O_2_ in 0.1 M, pH = 7.5 PBS. All electrochemical experiments were carried out at room temperature and without stirring.

## 3. Results and Discussion

### 3.1. Characteristics of the Au–rGO Nanocomposite

**Figure 1:**
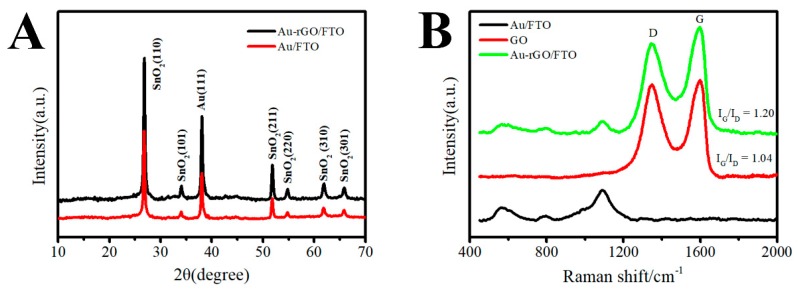
Typical XRD patterns (**A**) of 3D Au-rGO/FTO and Au/FTO, and Raman spectra (**B**) of GO/FTO, Au/FTO and 3D Au-rGO/FTO.

From Figure 1A, the XRD patterns of the 3D Au-rGO/FTO and 3D Au/FTO can be observed to be similar. The diffraction peaks of Au could be found in 38.2°, which corresponds to the (111) crystal plane according to the PDF 04-0784 card. In addition, the peaks in 26.6°, 33.9°, 51.8°, 54.7°, 61.9° and 65.9° could be indexed according to tetragonal SnO_2_ (PDF 41-1445 card). However, the diffraction signal of the rGO was indiscoverable in the XRD of 3D Au-rGO/FTO, for the reason that the rGO was well distributed in the sample. Raman spectroscopy were employed to characterize the composition in a facile and comprehensive way. In the Raman spectra (Figure 1B), the peak located in 1095 cm^−1^, belonging to Au, could be found in the spectra of 3D Au-rGO/FTO and Au/FTO, whereas, the D and G peak of GO were well-defined in the spectra of GO and 3D Au-rGO/FTO, which were attributed to the in-plane A_1g_ zone-edge mode and the E_2g_ mode of GO, respectively [18]. Meanwhile, the ratios between the intensity of the G peak and the D peak (I_G_/I_D_) were estimated from the Raman spectra. The calculation results indicated that I_G_/I_D_ in 3D Au-rGO/FTO (I_G_/I_D_ = 1.20) was higher than that in GO (I_G_/I_D_ = 1.04), which prove that GO was transformed into rGO during the preparation process. And Figure S1 further demonstrated this result. A strong reduction peak was observed at −1 V in the CV of Au-rGO/FTO (Figure S1), which indicated that GO deposited on the FTO surface was reduced. In summary, the data of XRD and Raman spectra given in Figure 1 confirm that 3D Au-rGO/FTO contained Au and rGO, and the GO was reduced during the electrochemical procedure.

**Figure 2:**
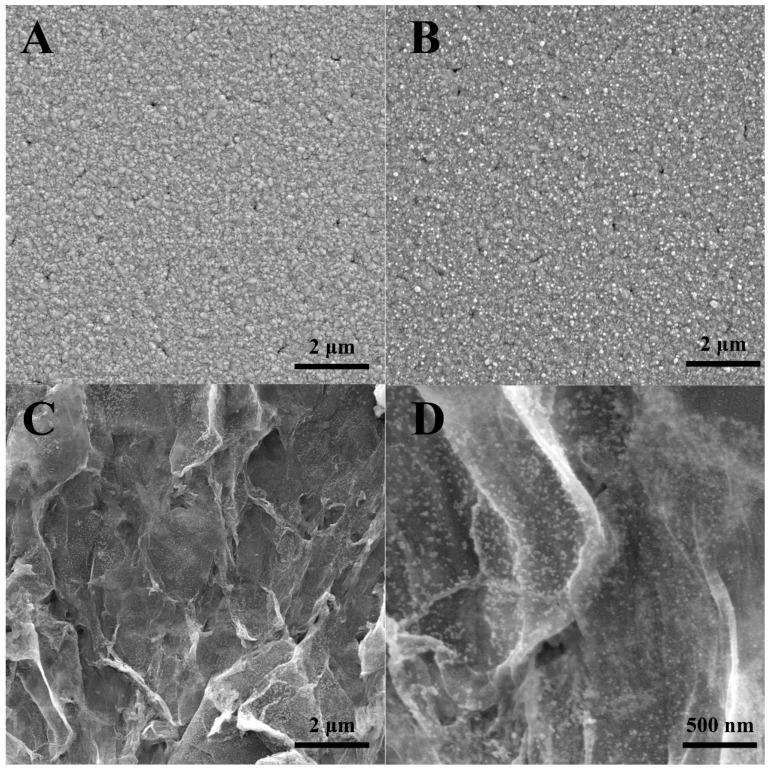
SEM images of FTO (**A**), Au/FTO (**B**), 3D Au-rGO/FTO (**C**) and higher magnification of 3D Au-rGO/FTO (**D**).

Figure 2 displays the SEM images of Au/FTO and 3D Au-rGO/FTO. Compared with the SEM image of FTO (Figure 2A), the AuNPs (Figure 2B) had been deposited on the FTO via the CV procedure. Figure 2B indicated that AuNPs were well-dispersed onto the FTO substrate in a high density. Figure 2C presents the SEM image of 3D Au-rGO/FTO which suggests that when GO was included in the electrolytes, the 3D Au-rGO could be electrodeposited on FTO via a one-step synthesis. Subsequently, the as-prepared AuNPs in the electrolytes with GO become smaller than that without GO, because GO could reduce the growth rate of AuNPs through influencing the diffusion of AuCl_4_^−^ on the surface of FTO. The SEM of 3D Au-rGO/FTO with higher magnification is profiled in Figure 2B. As could be seen, GO was well-dispersed with the assistance of AuNPs and a three-dimensional network structure was constructed with wrinkle-like rGO and AuNPs which was beneficial to the transfer of electrons in electrochemical analysis.

**Figure 3:**
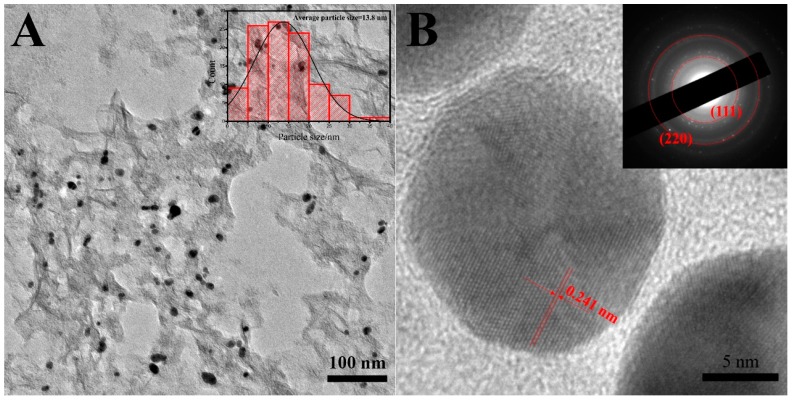
TEM images (**A**) of 3D Au-rGO and inset shows the particle size distribution, HRTEM images (**B**) of Au and inset shows the SAED pattern.

TEM was performed to investigate the morphology of 3D Au-rGO/FTO in detail. The TEM micrographs of 3D Au-rGO (Figure 3A) confirmed that AuNPs were well-distributed over the rGO and the average particle size of AuNPs were approximately 13.8 nm in diameter (from the inset in Figure 3A). AuNPs with small size were obtained, because the Cl^−^ in the electrolyte could complex with Au^3+^ to accelerate the dissolution of Au in large amounts at the high potential, which hindered the further growth of AuNPs.

The HRTEM image (Figure 3B) also exhibited crystalline lattice fringes of AuNPs in 3D Au-rGO with an interplanar distance of 0.241 nm, which can be assigned to the (111) plane of Au (PDF 04-0784 card). The selected area electron diffraction (SAED) pattern (inset of Figure 3B) exhibited two distinct concentric rings corresponding to the (111) and (220) planes of Au which were in agreement with the diffraction pattern of Au (PDF 04-0784 card).

### 3.2. Electrochemical Behavior on the Modified Electrodes

**Figure 4:**
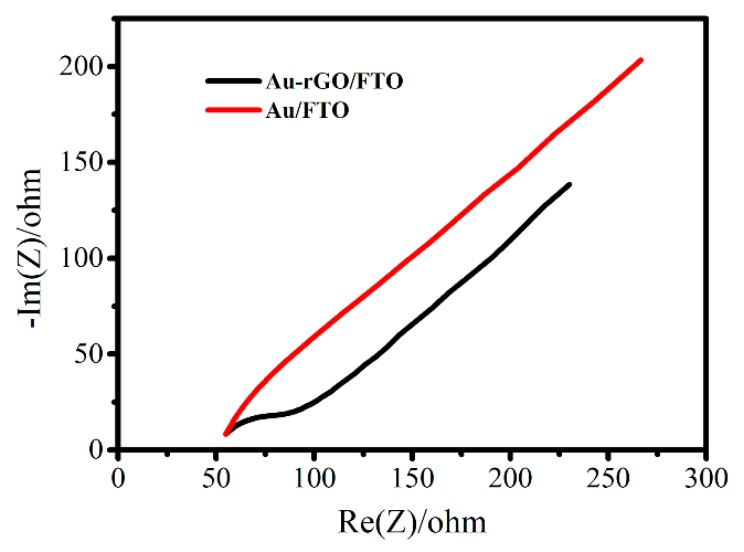
EIS of 3D Au-rGO/FTO (black line) and Au/FTO (red line) in 5 mM K_3_Fe(CN)_6_.

Electron transfer kinetics of different modified electrodes were investigated by EIS in solutions containing 5 mM K_3_Fe(CN)_6_. The diameter of the semicircle in the high frequency range implies the electron transfer resistance (R_ct_), which characterized the electron transfer kinetics of the electrochemical reaction. A comparison of the EIS of 3D Au-rGO/FTO and Au/FTO (Figure 4) showed that the semicircle diameter of Au/FTO was larger than that of 3D Au-rGO/FTO, which suggested faster electron transfer kinetics of 3D Au-rGO/FTO than Au/FTO. The above results could be explained as follows: rGO, with its excellent electrical conductivity properties and the structure of the composite could provide a conductive network to enhance the transfer of electrons in electrochemical reactions.

In order to estimate the electroactive surface area ($A_{e}$) of electrodes, CVs in different scan rates were performed in the solution with the presence of K_3_Fe(CN)_6_. The electroactive surface area was calculated based on the Randles-Sevcik equation [19]:

(1) $$I_{p}=2.69\times 10^{5}A_{e}n^{3/2}D_{0}^{1/2}v^{1/2}C_{0}$$

where $I_{p}$ is the redox peak current, *n* is the number of electrons participating in the redox reaction, which is taken as 1, $D_{0}$ is the diffusion coefficient of the Fe(CN)_6_^3−^ species, which is taken (6.70 ± 0.02) × 10^−6^ cm^2^ s^−1^ [19], v is the scan rate, $C_{0}$ is the concentration of the target molecules in the solution, which is taken 5 mM.

**Figure 5:**
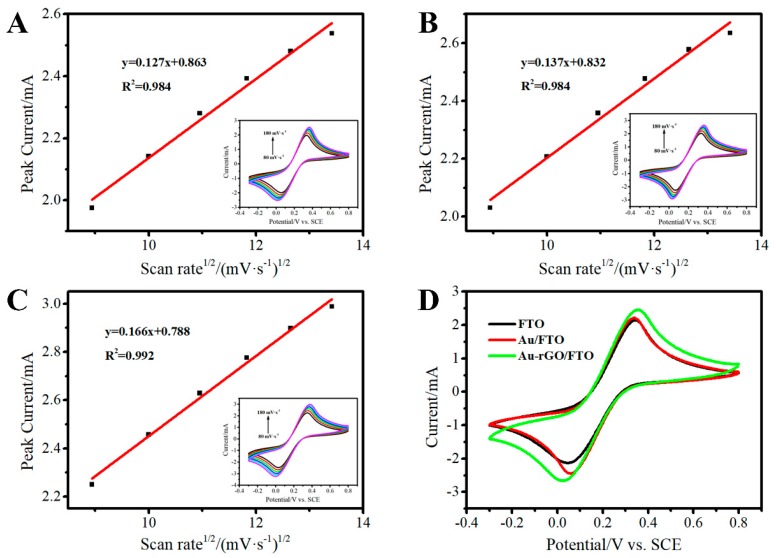
Peak current-scan rate^1/2^ responses of the FTO (**A**), Au/FTO (**B**), 3D Au-rGO/FTO (**C**) in K_3_Fe(CN)_6_ (insets show the CVs of the corresponding electrode at different scanning rates in K_3_Fe(CN)_6_), CVs of FTO, Au/FTO and 3D Au-rGO/FTO recorded in K_3_Fe(CN)_6_ (**D**).

The calibration curves of the oxidation peak currents versus the square of the scan rate of FTO (Figure 5A), Au/FTO (Figure 5B) and 3D Au-rGO/FTO (Figure 5C) were obtained from the CV results at different scan rates. As could be ascertained from the slope, that the electroactive surface area of 3D Au-rGO/FTO (0.075 cm^2^) was increased by 21% compared that of Au/FTO (0.062 cm^2^). However, the electroactive surface area of Au /FTO was only increased by 7% compared that of FTO (0.058 cm^2^). The CV test in 1 M NaOH (Figure S2) was used to estimate the actual area of 3D Au-rGO/FTO. According to the calculation results, the actual area of 3D Au-rGO/FTO was much larger than the active area of the electrode, because rGO was well dispersed on the surface of the FTO electrode and formed a large number of folds, which were mainly used for the construction of the three-dimensional network structure and promoted the dispersion of AuNPs. This signified that the usage of rGO was an important factor to increase the electroactive surface area due to the large specific surface area of rGO and the good dispersion of Au on the surface of rGO.

The number of active sites ($n_{activity}$) is a critical parameter to explain the electrochemical activity of an electrode or material. It could be estimated via the CVs by the following equation [20,21]:

(2) $$n_{activity}=Q/nF$$

where Q is the absolute components of the voltammetric charges; F is the Faraday constant (F), which is taken 96,485 C/mol, and *n* is the number of electrons transferred.

CVs of different electrodes are displayed in Figure 5D. Accordingly, the values of $n_{activity}$ of 3D Au–rGO/FTO, Au/FTO, and FTO were estimated as 79.8 nmol, 74.62 nmol, and 66.33 nmol, respectively, in terms of the Equation (2). This suggested that the number of active sites become more after the modification with Au and 3D Au-rGO. The result of the CVs analysis could imply that the electrochemical activity of the FTO was enhanced after modification with Au or 3D Au-rGO. and 3D Au–rGO/FTO has more active sites due to the large specific surface area of rGO and the good dispersion of Au on the surface of rGO.

### 3.3. Electrochemical Nitrite Sensor

#### 3.3.1. Electrochemical Behavior of Nitrite at Different Electrodes

**Figure 6:**
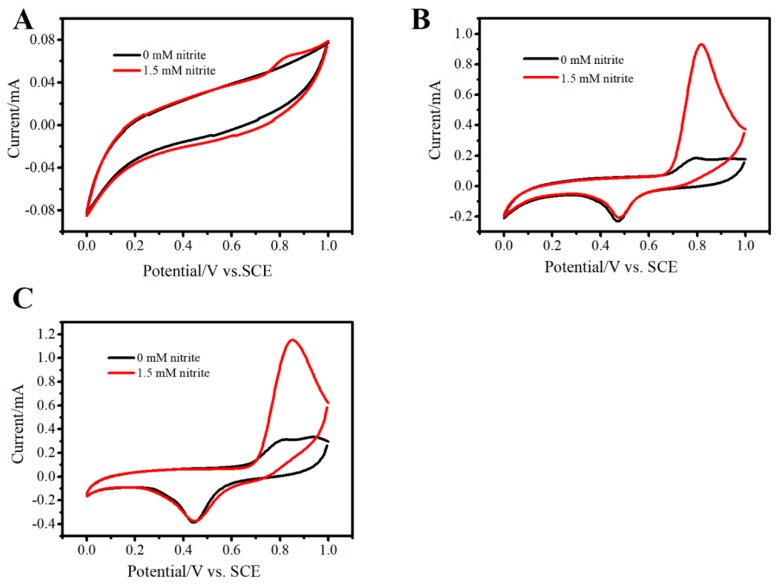
CVs of FTO (**A**), Au/FTO (**B**), 3D Au–rGO/FTO (**C**) in the 0 mM nitrite (black line) and 1.5 mM nitrite (red line) in PBS. Scan rate: 100 mV/s.

CVs of FTO (Figure 6A), Au/FTO (Figure 6B), 3D Au–rGO/FTO (Figure 6C) in 0 mM nitrite (black line) and 1.5 mM nitrite (red line) in pH 7.5 PBS were investigated. As can be seen, an irreversible oxidation peak, ascribed to the oxidation of nitrite, could be observed at 0.789 V (Au/FTO) and 0.815 V (3D Au-rGO/FTO) after adding nitrite into the solution, which verified the electrochemical activity toward nitrite. The irreversible oxidation peak of FTO was weak. Based on the CV, the increase of current in 3D Au-rGO/FTO (0.842 mA) was larger than that of Au/FTO (0.748 mA), demonstrating the sensitivity of 3D Au-rGO/FTO toward nitrite was higher than that of Au/FTO.

In order to explicate the result from CVs of Au/FTO, 3D Au–rGO/FTO in the absence and presence of nitrite, the values of catalytic rate constant ($k_{cat}$) of 3D Au–rGO/FTO and Au/FTO for nitrite were evaluated by chronoamperometry and calculated according to the following equation [22]:

(3) $$I_{C}/I_{L}=\pi^{1/2}{\left(K_{cat}C_{0}t\right)}^{1/2}$$

where $I_{C}$ is the catalytic current of the 3D Au–rGO/FTO in the presence of nitrite, $I_{L}$ is the limiting current in the absence of nitrite, $k_{cat}$ is the catalytic rate constant, $C_{0}$ is the nitrite concentration (1.5 mM), and t is time elapsed (s). From the slope of the $I_{C}/I_{L}$ vs. time^1/2^ plot, the value of $K_{cat}$ for a given concentration of nitrite can be computed.

**Figure 7:**
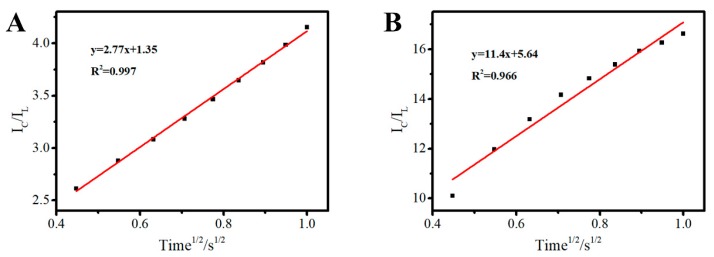
$I_{C}/I_{L}$-Time^1/2^ of Au/FTO (**A**), 3D Au–rGO/FTO (**B**). ($I_{C}$ is the catalytic current of the corresponding electrode in the presence of nitrite, $I_{L}$ is the limiting current in the absence of nitrite).

The calibration curves of $I_{C}/I_{L}$ versus the square of time based on chronoamperometry with Au/FTO (Figure 7A) and 3D Au-rGO/FTO (Figure 7B) are given in Figure 7. 3D Au-rGO/FTO with a slope of 11.4 and Au/FTO with a slope of 2.77 are presented in Figure 7. In another word, the plot of 3D Au-rGO/FTO is 4.1 times than the plot of Au/FTO, which implied the $K_{cat}$ of 3D Au-rGO/FTO (27.6 mM^−1^ s^−1^) is about 17 times than the $k_{cat}$ of 3D Au-rGO/FTO (1.63 mM^−1^ s^−1^). Therefore, the higher sensitivity of 3D Au-rGO/FTO toward nitrite than that of Au/FTO was attributed to the faster electrochemical reaction.

To sum up the analysis described above, the faster electrochemical reaction of 3D Au-rGO/FTO was in virtue of the following reasons: on one hand, the conductive network consisting of rGO could speed up the chemical reaction rate through enhancing the transfer of electrons in the electrochemical reaction; on the other hand, AuNPs could be well dispersed on the surface of FTO with the assistance of rGO to expose more electroactive surface area.

#### 3.3.2. Electrochemical Mechanism of Nitrite Oxidation at the Au-rGO/FTO

**Figure 8:**
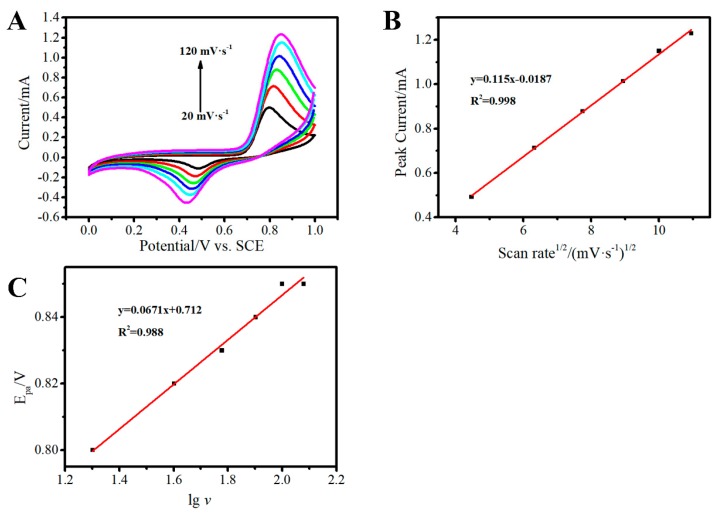
CVs of the 3D Au-rGO/FTO in PBS containing 1.5 mM nitrite at scan rates from 20 to 120 mV s^−1^ (**A**), the plots of anodic peak currents to the square root of scanning rates (**B**), anodic peak potentials versus lgv (**C**).

To explore the process of oxidation of nitrite, CVs in different rate were investigated in the solution containing nitrite, and the data were given in Figure 8A. Through the relationship analysis between anodic peak currents and scanning rates, it can be found that the anodic peak currents increased linearly with the square root of scanning rates (Figure 8B), implying that the oxidation for nitrite in Au–rGO/FTO was controlled by a diffusion process.

In addition, the linear relationship between anodic peak potential $E_{pa}$ and lgv (v is scan rate) had been investigated to calculate electron transfer number (*n*) as following Laviron’s equation [15]:

(4) $$E_{pa}=E^{0^{\prime }}+2.30\left(\frac{RT}{\left(1-\alpha \right)nF}\right)\mathrm{lg}v$$

where *α* is the electron transfer coefficient, n is the number of electron transferred, $E^{0^{\prime }}$ is the formal potential, v is the scan rate.

The plots of anodic peak potentials versus lgv are shown in Figure 8C. According to Equation (4), the electron transfer number (n) was estimated as 2 based on the slope of calibration curves when α is taken 0.5. Thus, the mechanism of nitrite oxidation could be described by Equation (5) [15,23], which could be divided into two steps reaction (Equations (6) and (7)) [15,23].

NO_2_^−^ + H_2_O → NO_3_^−^ + 2H^+^ + 2e^−^ (5)

2NO_2_^−^ ↔ 2NO_2_ + 2e^−^ (6)

2NO_2_ + H_2_O → NO_3_^−^ + NO_2_^−^ + 2H^+^ (7)

#### 3.3.3. Effect of Solution pH

**Figure 9:**
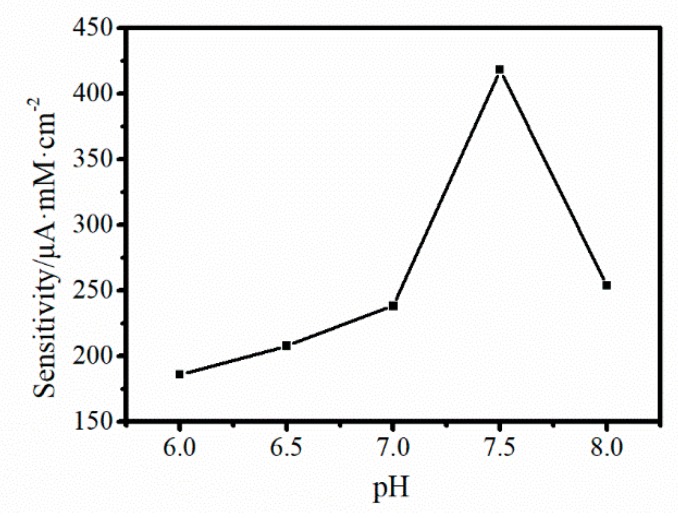
The sensitivity of 3D Au-rGO/FTO to nitrite against pH.

According to the Equation (5), H^+^ was one of reactants in the oxidation of nitrite, so the electrochemical reaction will be affected by the concentration of H^+^. In another word, pH was a vital factor to change the rate of oxidation of nitrite. Hereby, sensitivity for detection nitrite was surveyed (Figure 9) in order to optimize the testing condition for detection of nitrite. As could be seen in Figure 9, the sensitivity was increased with the rise of the pH from 6 to 7.5 and decreased at the pH 8, and the maximum sensitivity was obtained at pH 7.5. Therefore, the pH 7.5 was selected as the optimum pH value in our experiments.

#### 3.3.4. Determination of Nitrite on the 3D Au-rGO/FTO

**Figure 10:**
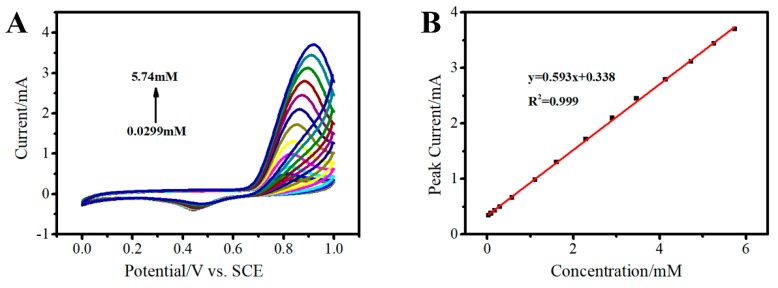
CVs of the 3D Au-rGO/FTO in PBS containing different concentrations of nitrite from 0.0299 to 5.74 mM (**A**); the relation between oxidation current versus the concentration of nitrite (**B**).

To probe the property of 3D Au-rGO/FTO toward the detection of nitrite, CVs were implemented for the quantitative detection of nitrite on the as-prepared 3D Au-rGO/FTO. As shown in Figure 10A, under the optimal conditions, the oxidation peak current of nitrite increased with its concentration from 0.0299 mM to 5.74 mM. As described in Figure 10B, the linear regression equation was expressed as I_pa_ = 0.593 C + 0.338, R^2^ = 0.999 (C = 0.0299–5.74 mM). The detection limit is found to be 0.0121 mM (S/N = 3) and the sensitivity is 419 μA mM^−1^ cm^−2^. Compared with the reported results (Table 1), the as-prepared 3D Au-rGO/FTO exhibited a wider linear range and a higher sensitivity than most of the results listed in Table 1, indicating that the nitrite could be detected using 3D Au–rGO/FTO in a wider linear range and with higher sensitivity. This phenomenon occurred because the one-step electrodeposition in the NaCl electrolyte could reduce the adsorption of impurity ions and surfactant on the surface of the material, and this could form a clean interface. The clean interface facilitated the exposure of the active site, which made the electrode more active and exhibit better detection performance. In addition, the results of stability, reusability and interference studies (Figure S6) toward nitrite are given in the Supplemental Information, which show the good stability, reusability and anti-interferent capability.

### 3.4. Electrochemical H_2_O_2_ Sensor

#### 3.4.1. Electrochemical Behavior of H_2_O_2_ at Different Electrodes

**Figure 11:**
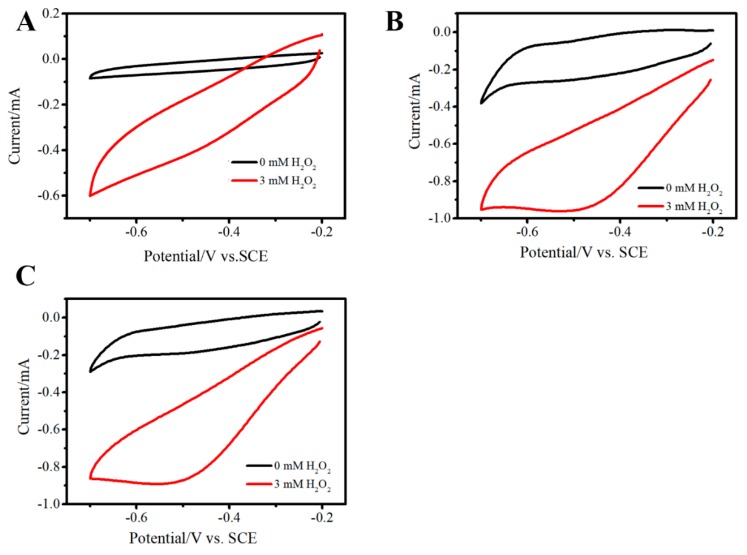
CVs of FTO (**A**), Au/FTO (**B**), 3D Au–rGO/FTO (**C**) in 0 mM H_2_O_2_ (black line) and 3 mM H_2_O_2_ (red line) in PBS. Scan rate: 100 mV/s.

The electrochemical activity of the 3D Au-rGO/FTO toward H_2_O_2_ reduction was investigated. Figure 11A–C profile the CVs of FTO, Au/FTO and 3D Au-rGO/FTO, respectively, in the absence (black curves) and presence (red curves) of 3 mM H_2_O_2_ in PBS. As can be seen from Figure 11, when H_2_O_2_ was added into the solution, irreversible peaks of Au/FTO and 3D Au-rGO/FTO were obvious, ascribed to the reduction of H_2_O_2_. Irreversible peaks of FTO toward H_2_O_2_ were not observed. According to the literature [3], the mechanism for H_2_O_2_ electro-reduction can be expressed as follows:

H_2_O_2_ + e^−^ → OH_ad_ + OH^−^ (8)

OH_ad_ + e^−^ → OH^−^ (9)

2OH^−^ + 2H^+^ → 2H_2_O (10)

Furthermore, 3D Au-rGO/FTO responds much more to H_2_O_2_ than Au/FTO as the active surface area and active site number of the 3D Au-rGO complex were larger than those of Au.

In addition, Figure S3A (Supplementary Information) presents the cyclic voltammograms of H_2_O_2_ on 3D Au-rGO/FTO in PBS at different scan rates. The reduction peak current raised gradually with the increase of the scan rate accompanied while the cathodic peak become negative. As shown in Figure S3B (Supplementary Information), the cathodic peak current was linear with the square root of scan rate in the range of 20–120 mV s^−1^, being this characteristic of a diffusion-controlled electrode process.

The catalytic rate constant ($k_{cat}$) of 3D Au-rGO/FTO electrode and Au/FTO for H_2_O_2_ (Figure S4A,B, Supplementary Information) were estimated as 1.90 mM^−1^ s^−1^ and 1.12 mM^−1^ s^−1^ respectively, according to Equation (3). 

The pH of the solution was optimized to achieve improved detection results. The influence of the pH value on the sensitivity of H_2_O_2_ was evident in Figure S5 (Supplementary Information). As shown, pH 7.5 was the optimal condition for the electrochemical reduction of H_2_O_2_. The decrease of the current value on higher pH might be related to the self-degradation of H_2_O_2_ [32], so PBS at pH 7.5 was employed in all the subsequent experiments.

#### 3.4.2. Determination of H_2_O_2_ on 3D Au-rGO/FTO

**Figure 12:**
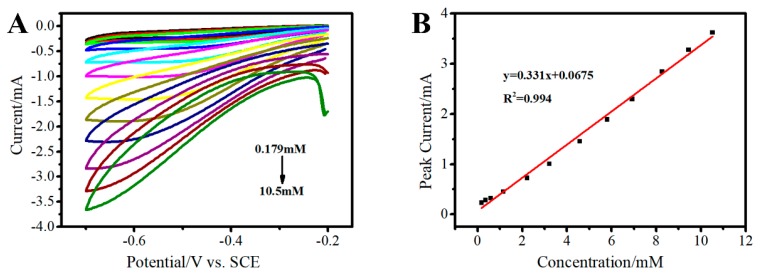
CVs of the 3D Au-rGO/FTO in PBS containing different concentrations of H_2_O_2_ from 0.179 to 10.5 mM (**A**). The relationship between oxidation current versus the concentration of H_2_O_2_ (**B**).

The detection of H_2_O_2_ using CVs based on the direct electrochemical reduction of H_2_O_2_ on 3D Au-rGO/FTO was performed (Figure 12A). As Figure 12B describes, the linear regression equations were expressed as I_pa_ = 0.331 C + 0.0675, R^2^ = 0.994 (C = 0.179–10.5 mM). The detection limit was found to be 0.0196 mM (S/N = 3) and the sensitivity was 236 μA mM^−1^ cm^−2^.

The property of 3D Au-rGO/FTO for the determination of H_2_O_2_ was better than most of the results displayed in Table 2. The obtained electrode showed a wider linear range and even lower detection limit, indicating that the obtained electrode had good electrochemical activity for the reduction of H_2_O_2_ due to the clean interface. Meanwhile, the results of stability, reusability and interference studies (Figure S7, Supplementary Information) toward H_2_O_2_, show the good stability, reusability and anti-interferent capability.

### 3.5. Simultaneous Electrochemical Detection of Nitrite and H_2_O_2_

For simultaneous electrochemical detection of nitrite and H_2_O_2_, a mixture of nitrite and H_2_O_2_ was used for the test by 3D Au-rGO/FTO. In the test, CVs were used and the voltage range was extended from −1 V to 1 V. According to the results (Figure S8, Supplementary Information), the recovery of nitrite and H_2_O_2_ in different concentrations was nearly 100% (Table 3). This indicated that detecting nitrite and H_2_O_2_ simultaneously by 3D Au-rGO/FTO was feasible. Stability and interference studies of the 3D Au-rGO/FTO are also shown in the Supplemental Information.

## 4. Conclusions

In summary, 3D Au-rGO/FTO with a clean interface and superb electrochemical activity, such as larger electroactive surface area, more active sites, and bigger catalytic rate constant than Au/FTO was prepared successfully via a one-step electrodeposition. This was because rGO with its excellent electrical conductivity properties and the structure of the composite could provide a conductive network to enhance the transfer of electrons in electrochemical reactions. The use of rGO was an important factor to increase the electroactive surface area due to the large specific surface area of rGO which was beneficial for the good dispersion of Au on the surface of rGO. According to the result, 3D Au-rGO/FTO can be applied in the detection of H_2_O_2_ with a sensitivity of 236 μA mM^−1^ cm^−2^ and nitrite with a sensitivity of 419 μA mM^−1^ cm^−2^ simultaneously, due to the conductive network consisting of rGO and the good dispersion of AuNPs on the surface of FTO with the assistance of rGO.

## Figures and Tables

**Table 1:** Comparison of different modified electrodes for nitrite determination.

Modified Electrode	Linear Range (mM)	Sensitivity (mA mM^−1^ cm^−2^)	Reference
Pt/MWCNTs/GC	0.004–2.4	0.41	[24]
Fe_3_O_4_/RGO/GCE	0.010–2.882	0.196	[25]
MOF-GNRs-50/ITO	0.100–2.500	0.094	[26]
Free-standing rGO/Acr paper	0.0004–3.6	0.40	[27]
Pd/Fe_3_O_4_/polyDOPA/RGO/GCE	0.0025–6.47	0.22	[28]
Pd/RGO/GCE	0.001–1.000	0.298	[29]
AuNPs-S-Gr/GCE	0.0125–0.681	0.208	[30]
TiO_2_/Ag/RGO/GCE	0.001–1.1	0.112	[31]
3D Au-rGO/FTO	0.0299–5.74	0.419	This work

**Table 2:** Comparison of different modified electrodes for H_2_O_2_ determination.

Modified Electrode	Linear Range (μM)	Sensitivity (μA mM^−1^ cm^−2^)	Reference
RGO-Au-PTBO/GCE	5.0–1077	63.39	[33]
AuPd@GR/ITO	5–11,500	186.86	[34]
RGO/Ag-Au/Cu_2_O/GCE	50–50,750	0.14	[35]
AuNPs-N-GQDs/GCE	0.25–13,327	186.22	[16]
RGO-PMS@AuNPs/GC	0.5–50,000	39.2	[36]
pore array-Ag/GCE	100–40,000	61	[37]
Au-TiO_2_/GR/GCE	10–200	151.5	[38]
Ni(II)-MOF/CNTs/GCE	10.0–51,600	117.14	[39]
POM-g-rGO/GCE	100–20,000	95.5	[40]
3D Au-rGO/FTO	179–10,500	236	This work

**Table 3:** Simultaneous electrochemical detection of nitrite and H_2_O_2_ based on 3D Au-rGO/FTO.

Sample	Nitrite Concentration (mM)		H_2_O_2_ Concentration (mM)		Recovery (%)	
	Actual	Detectable	Actual	Detectable	Nitrite	H_2_O_2_
Sample 1	1.5	1.57	1.5	1.63	104	109
Sample 2	3.0	3.20	3.0	3.02	107	101
Sample 3	5.0	4.47	5.0	5.26	95	105
Sample 4	5.0	5.26	7.5	7.19	105	95

## Supplementary Materials

The following are available online at https://www.mdpi.com/1424-8220/19/6/1304/s1. Figure S1 CVs for the synthesis of Au-rGO/FTO and Au/FTO; Figure S2 CV of Au-rGO/FTO in 1M NaOH. Scan rate: 50 mV/s; Figure S3 CVs of the 3D Au-rGO/FTO in PBS containing 3 mM H_2_O_2_ at scan rates from 20 to 120 mV s^−1^ (A), the plots of anodic peak currents to the square root of scanning rates (B); Figure S4 $I_{C}/I_{L}$-time^1/2^ of Au/FTO (A), 3D Au–rGO/FTO (B). ($I_{C}$ is the catalytic current of the corresponding electrode in the presence of H_2_O_2_, $I_{L}$ is the limiting current in the absence of H_2_O_2_); Figure S5 The sensitivity of 3D Au-rGO/FTO to H_2_O_2_ against pH; Figure S6 Results of the interference study on the response of 100-fold KCl and NaCl, 10-fold H_2_O_2_, ascorbic acid, glucose and Uric acid; Figure S7 Results of the interference study on the response of 10-fold NaNO_2_, KCl and NaCl, equivalent concentration of ascorbic acid, glucose and Uric acid; Figure S8 CVs of the 3D Au-rGO/FTO in mixture containing PBS (0.1M, PH = 7.5) and different concentrations of nitrite and H_2_O_2_.
