# Supplementary material for: Simultaneous Electrochemical Detection of Nitrite and Hydrogen Peroxide Based on 3D Au-rGO/FTO Obtained Through a One-Step Synthesis
Source: Sensors (Basel). 2019 Mar 15;19(6):1304. doi: 10.3390/s19061304 (PMC6471323; doi:10.3390/s19061304)
Supplement: Supplementary file 1 [file sensors-19-01304-s001.pdf]

Supplemental Information

Simultaneous Electrochemical Detection of Nitrite and Hydrogen Peroxide Based on 3D Au-rGO/FTO Obtained Through a One-Step Synthesis

Chengcheng Li [1], Delun Chen [1], Yuanyuan Wang [2], Xiaoyong Lai [3], Juan Peng [3], Xiaohong Wang [1], Kexi Zhang [1,*] and Yang Cao [1,*]

1 State Key Laboratory of Marine Resource Utilization in South China Sea, College of Materials and Chemical Engineering, Hainan University, Haikou 570228, China; lichengcheng@hainu.edu.cn (C.L.); chendelun2014@163.com (D.C.); wangxiaohong@hainu.edu.cn (X.W.)

2 Laboratory of Tropical Biomedicine and Biotechnology, School of Tropical Medicine and Laboratory Medicine, Hainan Medical University, Haikou 571199, China; vivian1004@126.com

3 Laboratory Cultivation Base of Natural Gas Conversion, School of Chemistry and Chemical Engineering, Ningxia University, Yinchuan 750021, China; xylai@nxu.edu.cn (X.L.); pengjuan@nxu.edu.cn (J.P.)

* Correspondence: zhangkexi@hainu.edu.cn (K.Z.); cy507@hainu.edu.cn (Y.C.); Tel./Fax: +86-898-66259764 (K.Z. & Y.C.)

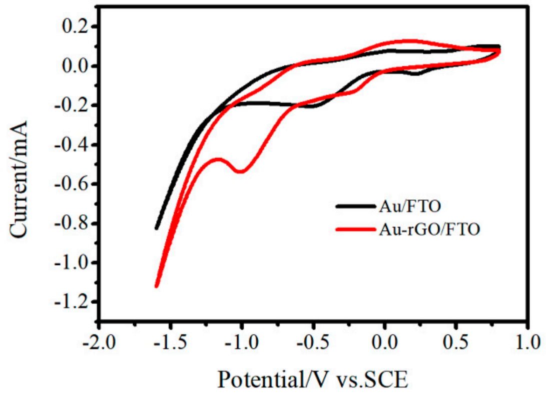

Figure S1. CVs for the synthesis of Au-rGO/FTO and Au/FTO

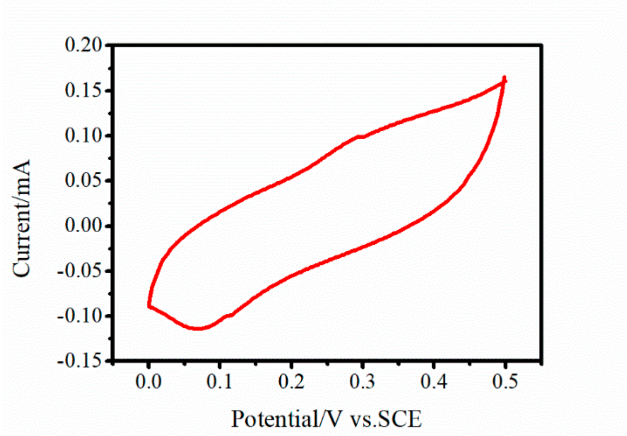

Figure S2. CV of Au-rGO/FTO in 1M NaOH. Scan rate: 50 mV/s

The capacitance values were calculated from the CV curves (Figure S2) according to the following Equation (S1)[1]:

$$C = \frac{1}{v(V_f - V_i)} \int_{V_i}^{V_f} I(V)\, dV \qquad \text{(S1)}$$

where ν is the scan rate (50mV s^{-1}), V_f and V_i are the integration potential limits of the voltammetric curve (V_f=0.5V, V_i=0.0V), and I(V) is the voltammetric discharge current (A). According to the calculation, C is 1024.4 μF.

Specific capacitance (C') of 3D Au-rGO/FTO was calculated based on the actual area (A) according to the following Equation (S2)[1]:

$$C' = \frac{C}{A} \qquad \text{(S2)}$$

For carbon materials, C' is 21 μF cm^{-2} [2], so the area of 3D Au-rGO/FTO could be estimated at 48.8 cm^2:

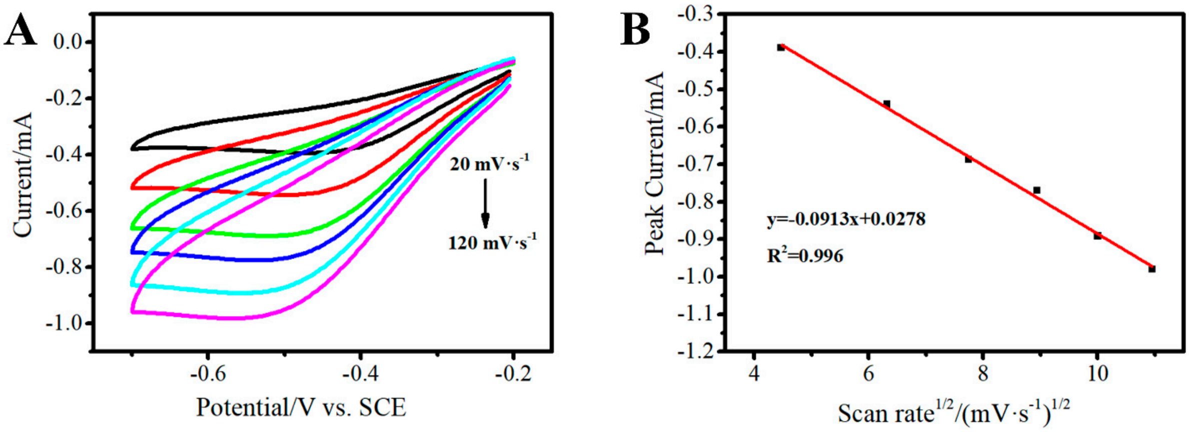

Figure S3. CVs of the 3D Au-rGO/FTO in PBS containing 3 mM H_2O_2 at scan rates from 20 to 120 mV s^{-1} (A), the plots of anodic peak currents to the square root of scanning rates (B)

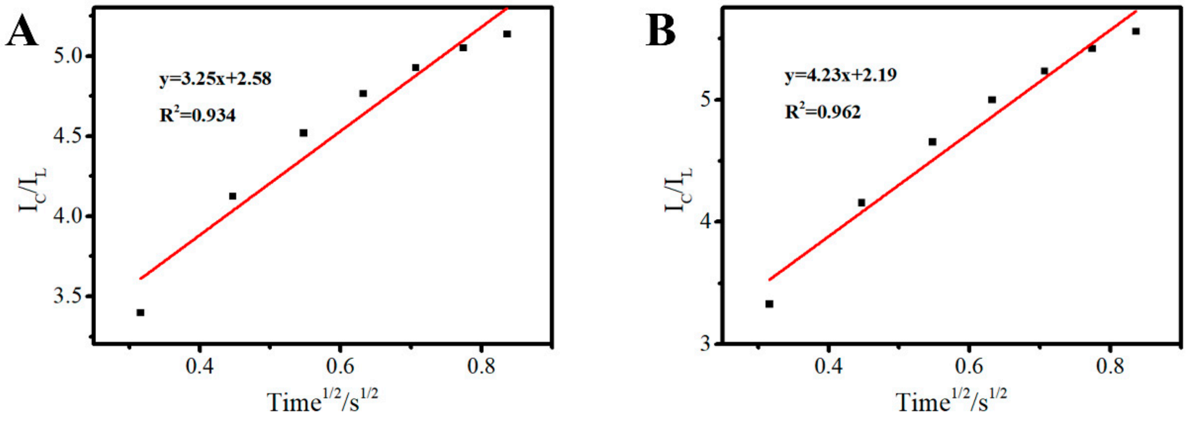

Figure S4. I_C/I_L-time$^{1/2}$ of Au/FTO (A), 3D Au–rGO/FTO (B). (I_C is the catalytic current of the corresponding electrode in the presence of H_2O_2, I_L is the limiting current in the absence of H_2O_2)

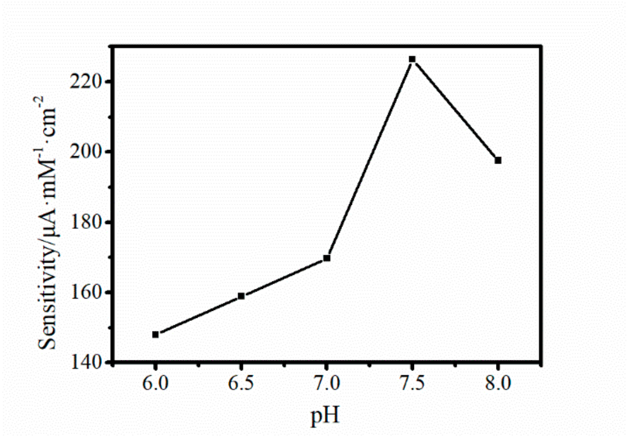

Figure S5. The sensitivity of 3D Au-rGO/FTO to H_2O_2 against pH

Stability, reusability and interference studies of the 3D Au–rGO/FTO

The stability of the modified electrode was also studied in the work by CV. It indicated a good stability in PBS containing 1.5 mM $NaNO_2$ solution with the relative standard deviation (RSD) of 1.6% (n = 5). It was found that the RSD of the reduction current was about 0.4% (n = 5) in PBS containing 0.3 mM H_2O_2. These clearly confirmed the excellent stability of 3D Au-rGO/FTO.

The reusability of five 3D Au-rGO/FTO electrodes was tested by CV. In pH = 7.5 PBS containing 1.5 mM $NaNO_2$ solution, the five electrodes showed good reusability with RSD of 7.0% (n = 5), and they also indicated a good reusability in pH=7.5 PBS containing 0.3 mM H_2O_2 with RSD of 5.9% (n = 5). The experimental results showed that 3D Au-rGO/FTO has good reusability.

The anti-interfering capability of 3D Au–rGO/FTO was evaluated. As Figure S6 shows, most of the species, such as NaCl and KCl in a 100-fold concentration, H_2O_2, ascorbic acid, glucose and uric acid in a 10-fold concentration, showed a little interference (lower than 5%) toward the determination of nitrite. All above results corroborated that 3D Au-rGO/FTO had a superb stability and good anti-interferent ability for the determination of nitrite.

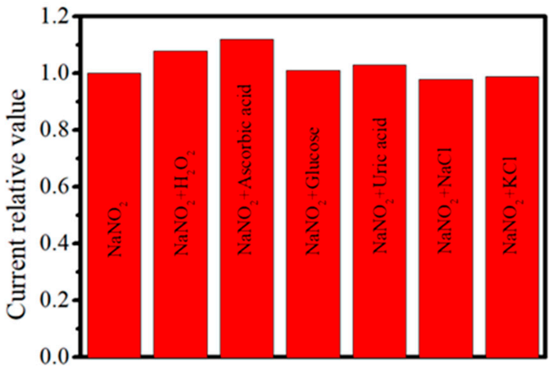

Figure S6. Results of the interference study on the response of 100-fold KCl and NaCl, 10-fold H_2O_2, ascorbic acid, glucose and uric acid

The electrochemical response of 3D Au-rGO/FTO for H_2O_2 in the presence of some interference, such as NaCl, KCl and $NaNO_2$ in a 10-fold concentration, ascorbic acid, glucose,

and uric acid in an equivalent concentration was shown in Figure S7. The interferences had little influence (lower than 5%) toward the determination of H_2O_2. The results verified that these substances did not cause an obvious interference for H_2O_2 detection, demonstrating a good selectivity of 3D Au–rGO/FTO.

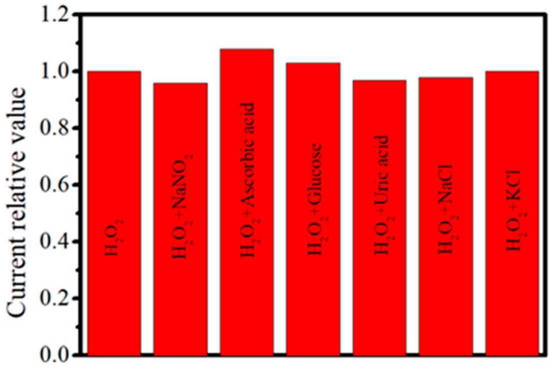

Figure S7. Results of the interference study on the response of 10-fold $NaNO_2$, KCl and NaCl, equivalent concentration of ascorbic acid, glucose and uric acid

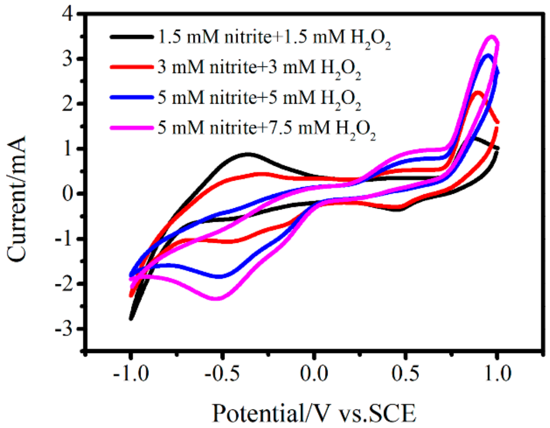

Figure S8. CVs of the 3D Au-rGO/FTO in mixture containing PBS (0.1M, PH = 7.5) and different concentrations of nitrite and H_2O_2

References

1. Shi, X.; Wu, Z.-S.; Qin, J.; Zheng, S.; Wang, S.; Zhou, F.; Sun, C.; Bao, X., Graphene-based linear tandem micro-supercapacitors with metal-free current collectors and high-voltage output. *Adv. Mater.* **2017**, *29*, 1703034.
2. Qi, J.L.; Wang, X.; Lin, J.H.; Zhang, F.; Feng, J.C.; Fei, W.-D., A high-performance supercapacitor of vertically-oriented few-layered graphene with high-density defects. *Nanoscale* **2015**, *7*, 3675–3682.
